# Machine learning and molecular subtype analyses provide insights into PANoptosis-associated genes in rheumatoid arthritis

**DOI:** 10.1186/s13075-023-03222-4

**Published:** 2023-12-01

**Authors:** Jing Li, Jun Cui, Li Wu, Ya-bing Liu, Qi Wang

**Affiliations:** 1 Department of Anesthesiology, Shanxi Provincial People’s Hospital (Fifth Hospital) of Shanxi Medical University, Taiyuan, China; 2 Department of Anesthesiology, The Hospital of Sinochem Second Construction Group Co, LTD, Taiyuan, China; 3 School of Basic Medical Sciences, Shanxi Medical University, Taiyuan, China; 4 Shanxi Key Laboratory of Big Data for Clinical Decision Research, Taiyuan, 030000 China

**Keywords:** Rheumatoid arthritis, PANoptosis, Molecular subtype, Machine learning, Treatment

## Abstract

**Background:**

PANoptosis represents a newly identified form of programmed cell death that plays a significant role in the autoimmune diseases. Rheumatoid arthritis (RA) is characterized by the presence of autoantibodies. Nevertheless, the specific biomarkers and molecular mechanisms responsible for the apoptotic characteristics of RA remain largely uninvestigated.

**Methods:**

We utilized 8 synovial tissue RA datasets. We selected genes associated with PANoptosis from the GeneCard database. By employing the limma, WGCNA, and machine learning algorithms we identified core genes. We utilized consensus clustering analysis to identify distinct PANoptosis subtypes of RA. Boruta algorithm was employed to construct a PANoptosis signature score. The sensitivity of distinct subtypes to drug treatment was verified using an independent dataset.

**Results:**

The SPP1 emerged as the significant gene, with its elevated expression in RA patients. We identified two PANoptosis RA subtypes. Cluster 1 showed high expression of Tregs, resting dendritic cells, and resting mast cells. Cluster 2 exhibited high expression of CD4 memory T cells and follicular helper T cells. Cluster 2 exhibited a higher degree of sensitivity towards immune checkpoint therapy. Employing the Boruta algorithm, a subtype score was devised for 37 PANoptosis genes, successfully discerning the subtypes (AUC = 0.794), wherein patients with elevated scores demonstrated enhanced responsiveness to Rituximab treatment.

**Conclusion:**

Our analysis revealed that SPP1 holds potential biomarker for the diagnosis of RA. Cluster 2 exhibited enhanced sensitivity to immune checkpoint therapy, higher PANoptosis scores, and improved responsiveness to drug treatment. This study offers potential implications in the realm of diagnosis and treatment.

**Supplementary Information:**

The online version contains supplementary material available at 10.1186/s13075-023-03222-4.

## Introduction

Rheumatoid arthritis is a prevalent chronic autoimmune disease characterized by synovial inflammation and joint cartilage destruction, resulting in joint deformity and disability [1–3]. Its global prevalence varies, with industrialized countries exhibiting higher rates, potentially attributable to both environmental and genetic factors. Given its chronic nature, rheumatoid arthritis poses challenges in treatment and is commonly likened to an enduring malignancy, imposing substantial economic burdens on individuals and society. Presently, prevailing clinical interventions for RA encompass the utilization of nonsteroidal anti-inflammatory drugs (NSAIDs) and disease-modifying antirheumatic drugs (DMARDs). In light of the intricate nature of this ailment, certain investigations have endeavored to investigate diverse amalgamated therapeutic approaches [4, 5]. Nevertheless, specific medications employed for treatment may engender diverse deleterious repercussions. Consequently, the management of RA patients necessitates customization according to distinct disease subtypes, achieved by identifying individual RA subtypes through biomarkers and implementing meticulous pharmacotherapeutic regimens.

An expanding body of empirical evidence indicates a mounting significance of cell death in diverse human ailments, encompassing cancer, autoimmune diseases, and neurodegenerative disorders [6–9]. PANoptosis, a phenomenon of inflammation-triggered programmed cell death, commonly referred to as cellular suicide, encompasses apoptosis, necrosis, and associated cell death mechanisms, thereby concurrently initiating multiple cell death pathways, such as apoptosis, necrosis, and pyroptosis. Consequently, it can be regarded as the most intricate manifestation of cell death documented thus far. The pathogenesis of RA involves various molecular mechanisms, including IL-17-mediated mitochondrial dysfunction leading to autophagic impairment and apoptosis of synovial fibroblasts [10]. Despite these findings, there is a notable dearth of studies investigating the role of PANoptosis in the pathogenesis of rheumatoid arthritis.

Hence, the objective of this study is to examine the biomarkers and molecular mechanisms linked to PANoptosis in patients with RA. Differential gene expression, weighted gene co-expression network analysis (WGCNA), and machine learning algorithms will be utilized to ascertain key diagnostic genes associated with PANoptosis, which will subsequently be validated in an independent cohort. Additionally, consensus clustering algorithms will be employed to identify potential subtypes of PANoptosis among RA patients, and a scoring system for PANoptosis will be developed to distinguish these subtypes, thereby investigating their responsiveness to pharmaceutical interventions and variations in the immune microenvironment. Our research findings have the potential to contribute to the identification of effective PANoptosis diagnostic biomarkers and guide treatment strategies for RA patients.

## Materials and methods

### Data collection and data preprocessing

We obtained four chip datas of synovial tissue from patients with RA and normal tissue from the GEO database, specifically GSE12021, GSE55235, GSE55457, and GSE77298. The GSE77298 being the sole training set. Additionally, we collected four drug treatment chip data of RA synovial tissues from GSE172188, GSE45867, GSE24742, and GSE15602 to investigate the response of different subtypes to drugs. The specific information for each chip is provided in Table 1. The original CEL files of all chip data were obtained and subjected to background adjustment, quantile normalization, and log transformation using the robust multi-chip average (RMA) algorithm, resulting in the generation of gene expression matrix files. To eliminate batch effects and combine the datasets, the "ComBat" function from the 'sva' package was employed. To identify the key diagnostic gene, SPP1, associated with PANoptosis-Related RA, we utilized GSE77298 as a training set and other datasets (GSE12021, GSE55235, GSE55457) as external independent validation sets to assess the differential expression and diagnostic performance of SPP1. In order to eliminate any potential batch effect, we combined four datasets (GSE12021, GSE55235, GSE55457, and GSE77298) to identify the pan-apoptotic RA subtype.

**Table 1:** The datasets employed in this study

Data set	Subjects	Experiment type	Platforms	Tissue	Drug
GSE12021	12 RA vs 9 HC	Expression profiling by array	GPL96\GPL97	Synovial	NA
GSE55235	10 RA vs 10 HC	Expression profiling by array	GPL96	Synovial	NA
GSE55457	13 RA vs 10 HC	Expression profiling by array	GPL96	Synovial	NA
GSE77298	16 RA vs 7 HC	Expression profiling by array	GPL570	Synovial	NA
GSE172188	10 RA	Expression profiling by array	GPL570	Synovial	Abatacept
GSE45867	10 RA	Expression profiling by array	GPL570	Synovial	Methotrexate
GSE24742	12 RA	Expression profiling by array	GPL570	Synovial	Rituximab
GSE15602	11 RA	Expression profiling by array	GPL570	Synovial	Adalimumab

### Weighted gene co-expression network analysis (WGCNA) for identifying RA-related core genes

The R package WGCNA was utilized to construct a co-expression network and ascertain co-expression modules associated with RA. Subsequently, the weighted adjacency matrix was transformed into a topological overlap matrix (TOM), and genes were hierarchically clustered based on dissimilarity (dissTOM = 1 − TOM) of the topological overlap. Modules exceeding a gene count of 50 were chosen employing the hierarchical clustering tree method. Genes exhibiting strong correlation with clinical features were extracted from these modules.

### Analysis of differentially expressed genes

The limma package [11] was employed to conduct an analysis of differentially expressed genes (DEGs) between synovial tissue affected by RA and normal tissue. Genes meeting the criteria of |Log2fold change|> 1 and adjusted *P*-value < 0.05 were selected for filtering purposes. To visualize the DEGs, a volcano plot and heatmap were utilized. By employing a screening criterion of a relevance score > 3 within the Genecards database, a total of 1324 panptosis-related genes (comprising 1313 apoptosis genes, 11 necrosis genes, and 31 pyroptosis genes) were identified using the search terms "apoptosis", "necroptosis", and "pyroptosis" [12]. To identify intersection genes, we took the intersection of module-specific genes obtained from WGCNA, DEGs, and apoptosis-related genes.

### Protein–protein interaction analysis of PANoptosis-related genes

The STRING database [13] was utilized to generate a protein–protein interaction (PPI) network for the intersecting genes, employing a minimum confidence score threshold of greater than 0.4. GeneMANIA, an online database, was employed to ascertain genes associated with a given set of input genes.

### Gene function enrichment analysis

The R package "clusterProfiler" [14] and the online analysis tool Metascape [15] were employed for conducting GO and KEGG pathway enrichment analyses on differentially expressed genes. Adjusted *p*-values < 0.05 were considered statistically significant.

### Identification of disease-related feature genes

Four machine learning algorithms, namely LASSO, SVM-REF, Boruta, and RF, were employed collectively to ascertain disease-related feature genes. LASSO, a regression analysis technique, was utilized for feature selection and regularization, with the objective of enhancing the predictive accuracy and interpretability of statistical models. SVM-RFE, an efficient feature selection approach, was employed to identify the optimal variables by eliminating feature vectors generated by SVM [16]. Boruta, a supervised classification feature selection method rooted in random forest, was employed to identify all pertinent features for a classification task. The random forest classifier is an ensemble learning algorithm that constructs a decision tree ensemble by utilizing randomly selected training data and feature subsets.

### Identification of PANoptosis-related RA subtypes

In order to investigate potential PANoptosis subtypes in patients with RA, we employed the "ConsensusClusterPlus" package for unsupervised clustering [17]. The clustering process was conducted with specific settings, including a maximum of six clusters (maxK = 6), the PAM clustering algorithm, and the Euclidean correlation method. To ensure clustering stability, we repeated the process for 1000 iterations. The evaluation criteria for each cluster involved the utilization of cumulative distribution function (CDF) values and the incremental area under the CDF curve. The reliability of the clustering results was subsequently confirmed through the application of principal component analysis (PCA). PCA was performed using the prcomp function.

### Immune infiltration analysis

In order to assess the variations in immune features among different PANoptosis subtypes in patients with RA, the CIBERSORT algorithm [18] was employed to quantify the levels of infiltration by 22 immune cell types. Additionally, Spearman correlation analysis was conducted to examine the relationship between feature genes and immune cells.

### Construction of PANoptosis score for RA patients

To establish a PANoptosis score for RA patients, first, the Pearson correlation analysis was employed to categorize the 68 differentially expressed PANoptosis genes. Furthermore, 68 differentially expressed PANoptosis gene values that were positively and negatively correlated with the cluster signature were termed as the signature gene A and B, respectively. Furthermore, the Boruta algorithm was employed for the dimension reduction of the signatures A (31 genes) and B (6 genes), and principal component 1 was extracted as the signature score by employing the PCA. The formula for calculating the feature score is as follows:

$$\mathrm{GeneScore}=\mathrm{PCA}1.\mathrm{A}-\mathrm{PCA}1.\mathrm{B}$$

The term GeneScore refers to the PANoptosis score, which offers a partial representation of the overall apoptosis pattern associated with the disease.

### Statistical analysis

All statistical analyses were performed using R version 4.2.0 software. The Mann–Whitney *U* test was used to compare the expression differences between two groups, and a *p*-value or adjusted *p*-value less than 0.05 was considered statistically significant.

## Results

### Weighted gene co-expression network construction and key module identification

**Fig.1:**
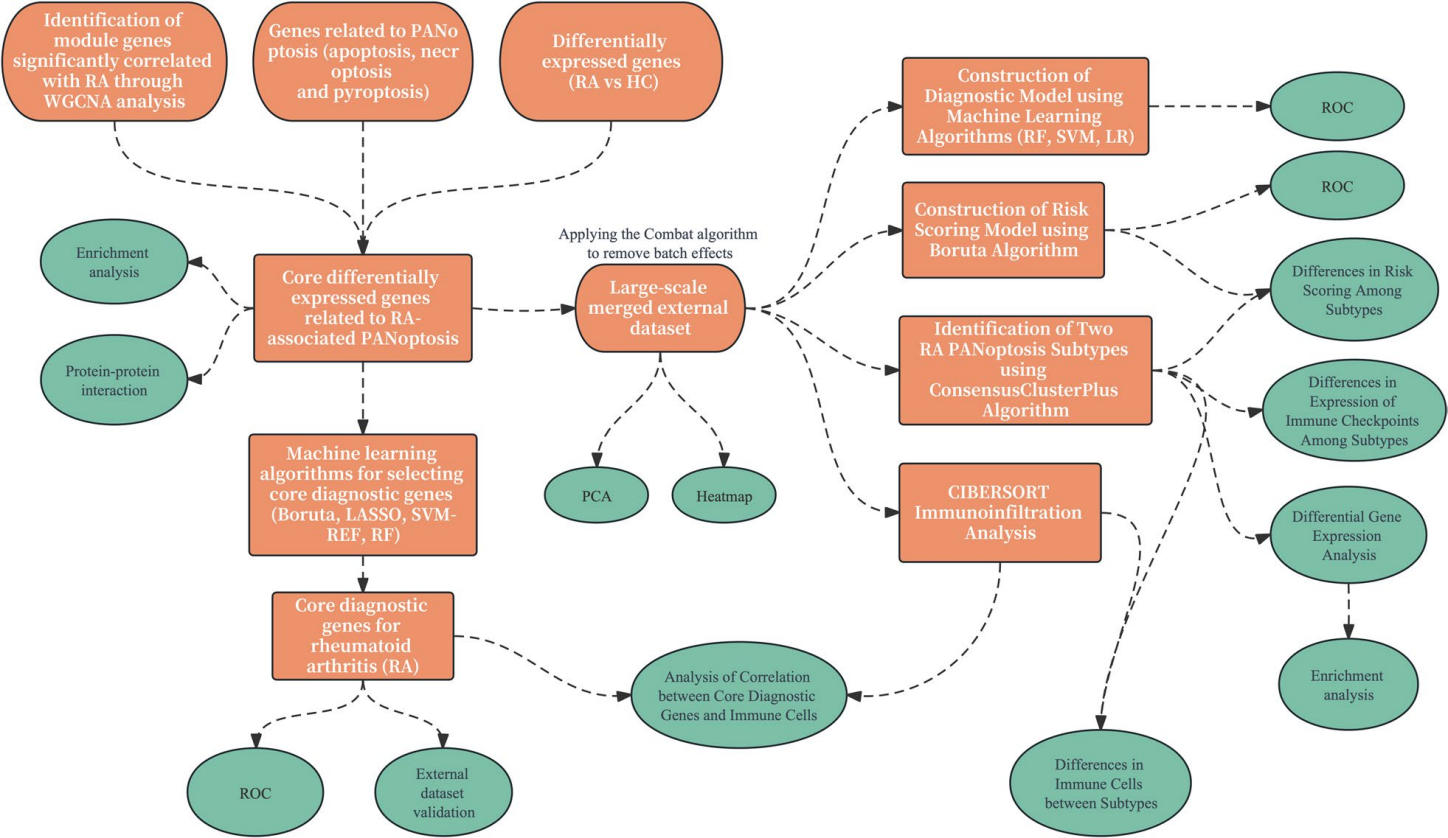
Flow diagram of the study

**Fig. 2:**
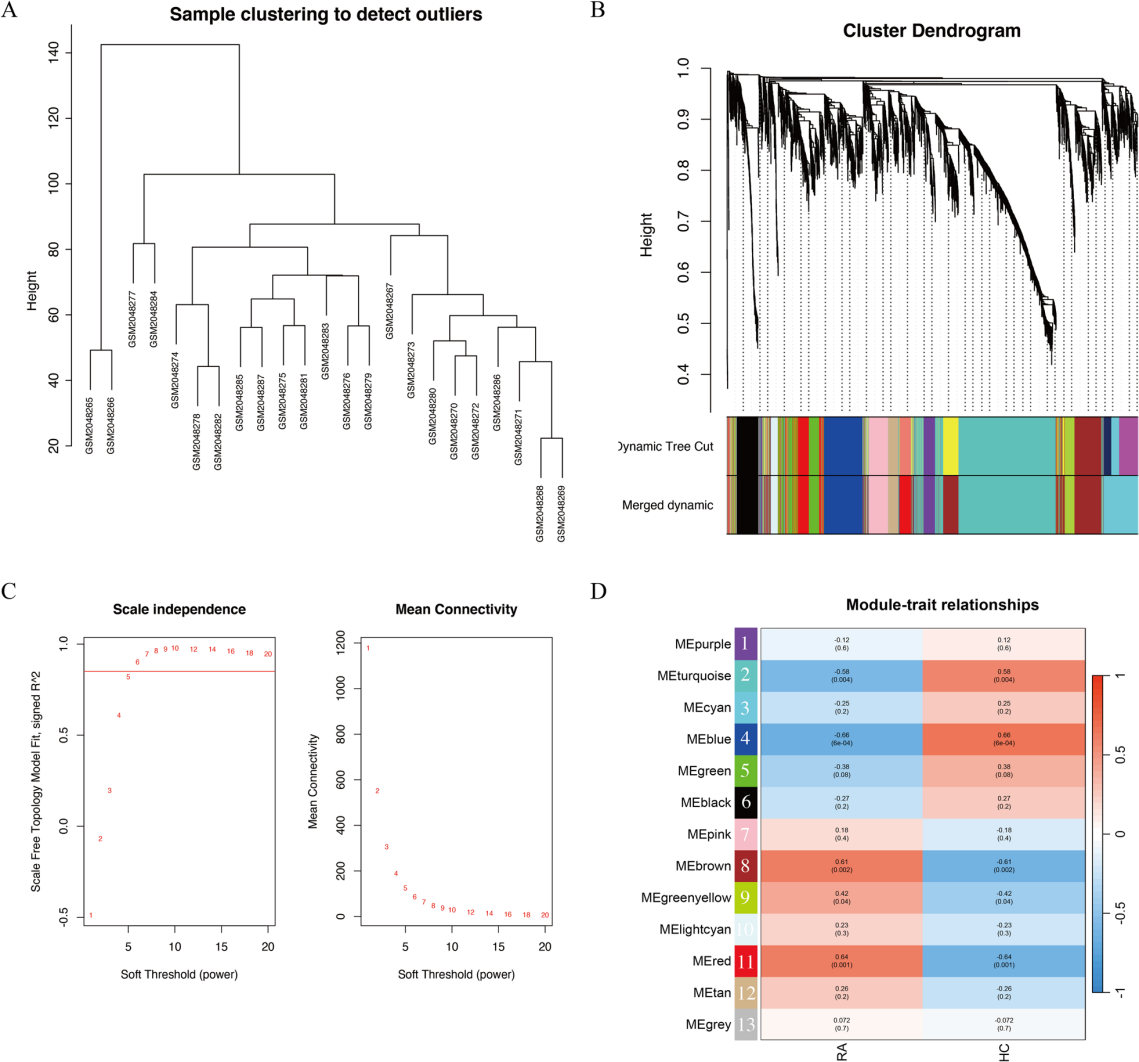
Construction of co-expression network modules. **A** Sample clustering tree diagram. **B** Genes with similar expression patterns were clustered, different colors are different gene clusters, grey modules represent genes not assigned to any of the modules. **C** Optimal soft threshold power. **D** Heatmap of module-trait correlations

An overview of the study can be found in Fig. 1. To identify module genes significantly associated with RA patients, a clustering analysis was conducted on a dataset (GSE77298) consisting of 23 samples. The results of the clustering analysis were visualized through a sample clustering tree (Fig. 2A,B). With help from the "pickSoftThreshold" function, a signed network was constructed and the module eigengene expression was calculated with the aid of the "blockwiseModules" function. The determination of the optimal soft threshold power, which exhibits higher average connectivity (Connectivity refers to the number of nodes directly connected by a node), was achieved by setting the threshold at 6 when the coefficient of determination (R^2^) exceeded 0.85 (Fig. 2C). Furthermore, a module-clinical feature correlation heatmap was generated, leading to the identification of a total of 13 modules. Notably, the red module (Model number = 11) exhibited a strong positive correlation with RA (*r* = 0.64, *p* = 0.001), while the blue module (Model number = 4) displayed a strong negative correlation with RA (*r* =  − 0.66, *p* = 6e − 04) (Fig. 2D). Therefore, we selected the genes from the red and blue modules for further analysis.

### Identification of differentially expressed PANoptosis genes in RA

**Fig. 3:**
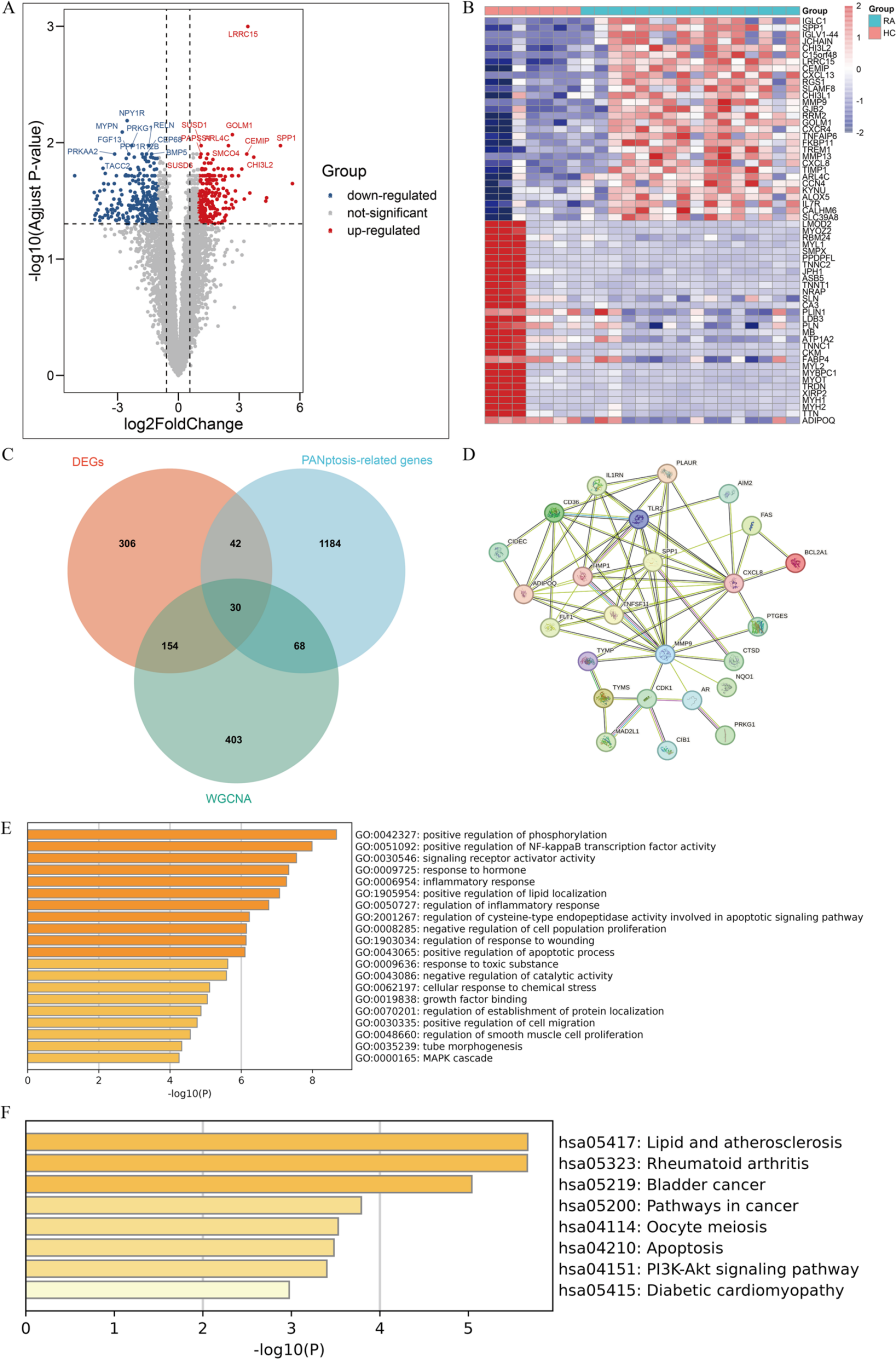
Identification of differentially expressed PANoptosis-related gene in RA patients. **A** Volcano plot of DEGs. **B** Heatmap of the DEGs. **C** Venn diagram of the intersection of DGEs, PANoptosis-related gene and WGCNA significant module genes. **D** Protein–protein interaction network of 30 Intersect Genes". **E** GO analysis of 31 intersect genes. **F** KEGG analysis of 30 intersect genes

Through differential analysis, we finally identified 265 upregulated genes and 267 downregulated genes in RA and HC tissues. The volcano plot and heatmap of differential genes are shown in Fig. 3A and B. To further screen for signature genes, we took the intersection of genes from the two highest correlated modules in WGCNA, DEGs, PANoptosis-related genes from Genecards, resulting in 30 "Intersect genes" (Fig. 3C). GO enrichment analysis revealed that the 30 intersect genes are mainly involved in positive regulation of phosphorylation, positive regulation of NF-kappaB transcription factor activity, positive regulation of lipid localization, and regulation of cysteine-type endopeptidase activity involved in apoptotic signaling pathway. KEGG enrichment analysis showed that the differential genes are mainly associated with lipid and atherosclerosis and PI3KAkt signaling pathway (Fig. 3E,F). PPI network analysis revealed strong correlations between SPP1, CXCL8, MMP9, and TIMP1 (Fig. 3D).

### Machine learning algorithms identify the target gene SPP1

**Fig. 4:**
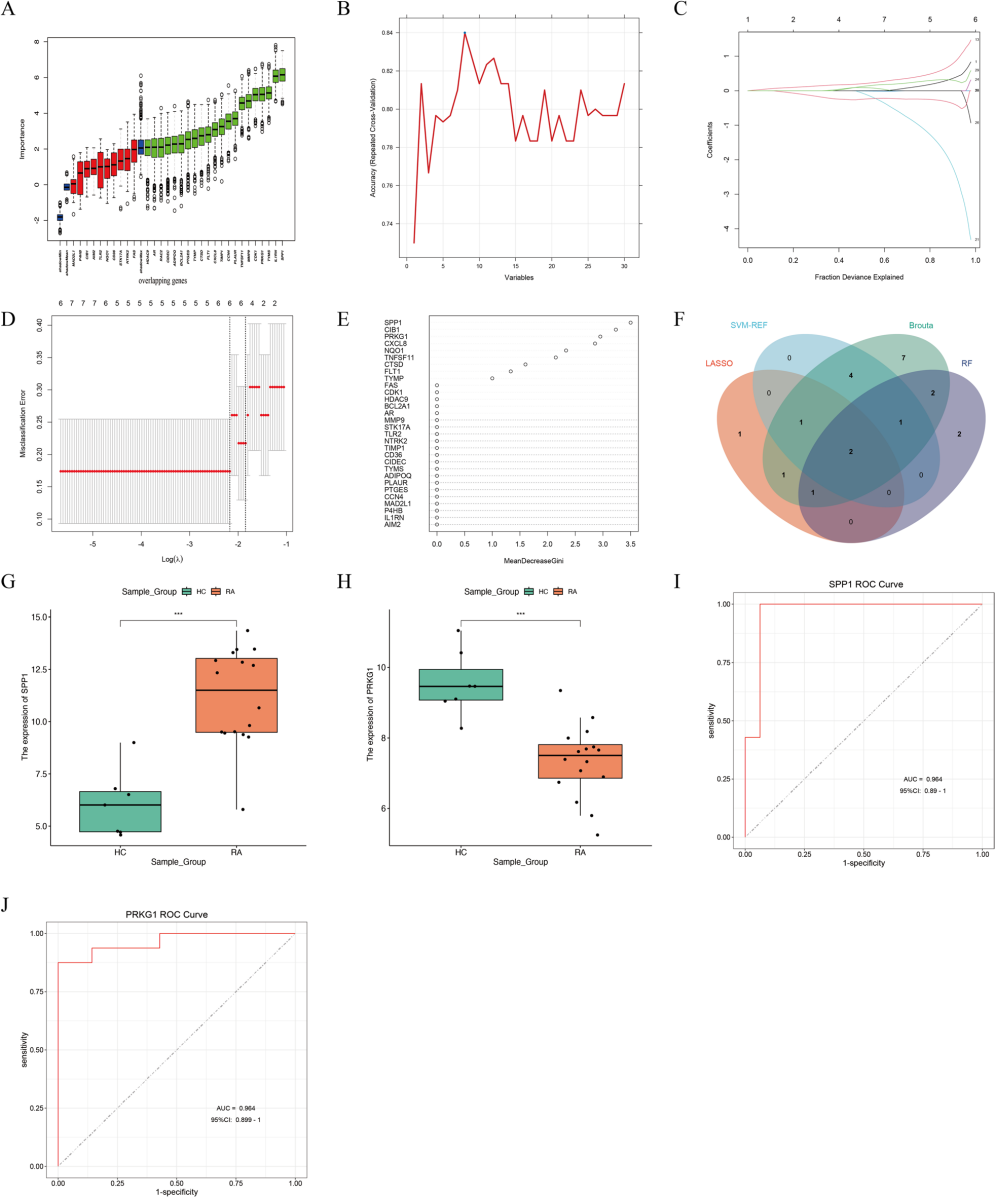
Selection of feature genes and determination of target SPP1. **A** Boruta selection of 19 feature genes with importance ranking. Green represents important genes selected by Boruta algorithm after dimensionality reduction, blue represents shadowMax value, that is, the threshold value of importance score, and red represents unimportant genes after dimensionality reduction by Boruta. **B** SVM-RFE selection of 8 feature genes. **C** Coefficients were calculated for each lambda. Each line represents a gene confidence value. **D** LASSO regression analysis of 6 genes. The horizontal axis represents the log value of the independent variable, while the vertical axis represents the partial likelihood deviance of the log value of each independent variable. **E** RF selection of 8 feature genes with importance ranking. **F** Venn plot of the overlapping genes identified through the four machine algorithms. **G** Expression of SPP1 in GSE77298. **H** Expression of PRKG1 in GSE77298. **I** ROC curve of SPP1 in GSE77298. **J** ROC curve of PRKG1 in GSE77298

To identify predictive factors for RA patients, we employed four machine learning algorithms to reduce the dimensions of 30 overlapping genes. The Boruta algorithm selected a total of 19 genes, with SPP1 ranking first in importance (Fig. 4A). The support vector machine method identified 8 genes as important biomarkers for RA (Fig. 4B). The selection of 8 genes as feature variables yielded the optimal prediction performance, with a model accuracy of 0.84. This outcome suggests that these 4 features can be considered as the most suitable subset for predicting RA. Using the LASSO algorithm, we discovered 6 genes that could serve as potential markers for RA (Fig. 4C,D). By applying the criterion of average reduction in mean decrease gini greater than 1, we employed random forests to select 8 genes, with SPP1 also ranking first in importance (Fig. 4E). By taking the intersection of the feature genes from the four machine learning algorithms, we obtained two feature genes, namely SPP1 and PRKG1 (Fig. 4F). In the training set, we analyzed the expression of these two genes, as evident from the box plots, where SPP1 exhibited upregulation in RA and PRKG1 showed downregulation, both with statistical significance (Fig. 4G,H). The receiver operating characteristic (ROC) analysis of the diagnostic effectiveness of the biomarkers revealed that both SPP1 (AUC = 0.964) and PRKG1 (AUC = 0.964) had high diagnostic value for RA and HC groups (Fig. 4I,J). Furthermore, we validated the expression of these two genes in three independent external datasets. In GSE55235, SPP1 expression was significantly elevated in RA compared to the normal group (*P* < 0.001). Although there was no significant difference in the expression of SPP1 between RA and HC groups in GSE12021 and GSE55457, there was an upward expression trend, indicating a good diagnostic value of this gene across the three datasets (Supplementary Figure S1). The gene PRKG1 was not detected in other datasets. Therefore, SPP1 was selected as the core gene for further analysis in this study.

### PANoptosis-related molecular subtypes of RA

**Fig. 5:**
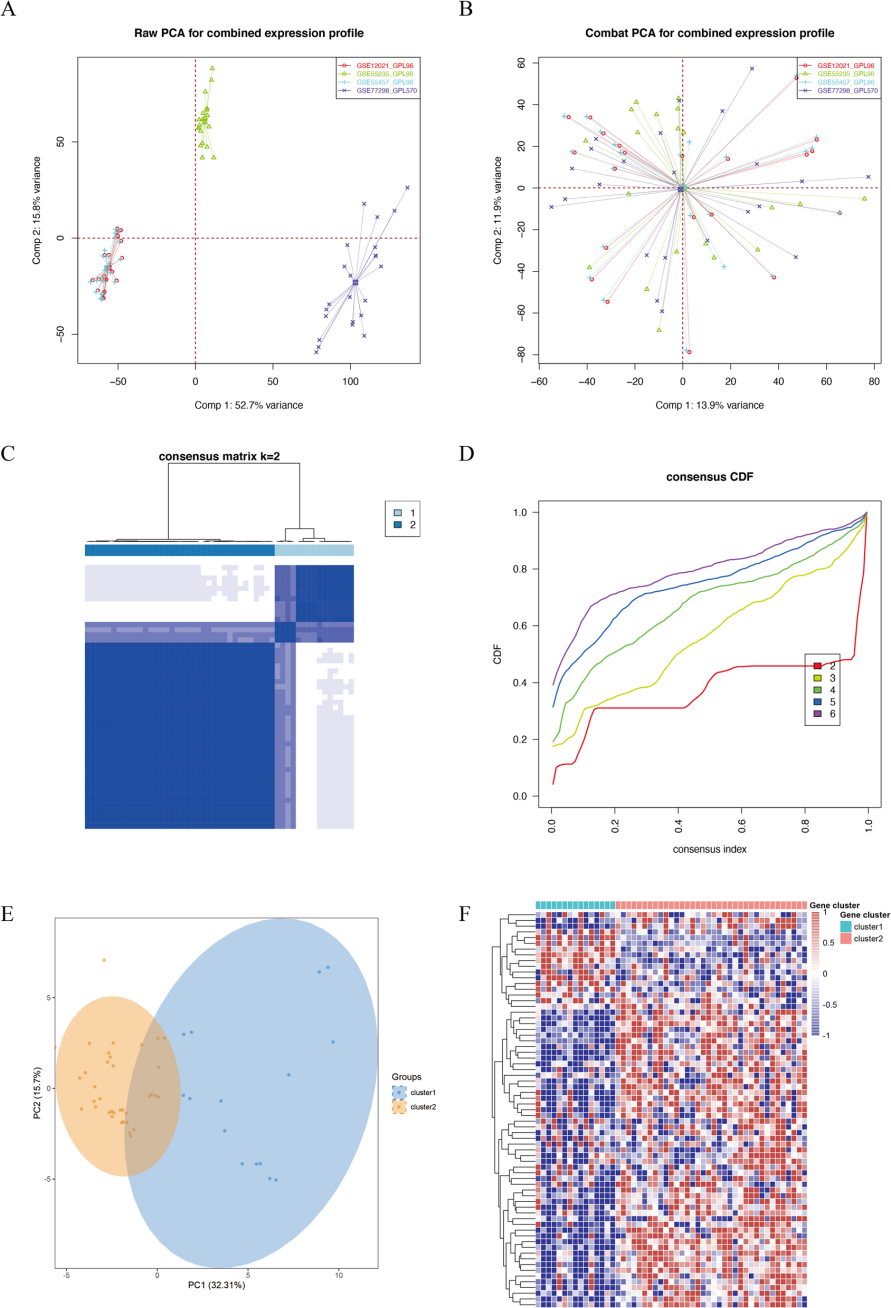
Consensus clustering of PANoptosis-related RA molecular subtypes based on 72 PANoptosis genes. **A** Scatter plots of PCA before removal of batch effects for the four data sets. **B** Scatter plots of PCA after removing batch effects by the combat function. **C** The consensus score matrix for RA samples when *k* = 2. **D** Consensus clustering cumulative distribution function (CDF) for *k* = 2–6, which can completely describe the probability distribution of a real random variable. **E** Principal components analysis showing the stability and reliability of clustering. **F** The distribution of 72 PANoptosis-related gene among two clusters

To identify PANoptosis-related molecular subtypes of RA, we merged four RA datasets and removed batch effects (Fig. 5A,B). Among the 72 differentially expressed PANoptosis genes in the merged expression profile, only 68 genes were expressed. We performed 1000 iterations using the 'ConsensusClusterPlus' R package, with the optimal number of clusters ranging from *k* = 2 to 6. Based on the cumulative distribution function (CDF) values and delta area, we recommend utilizing *k* = 2 clusters to ensure robust clustering results (Fig. 5C,D). Principal component analysis (PCA) plot and heatmap demonstrated significant differences between the two subtypes (Fig. 5E,F).

**Fig. 6:**
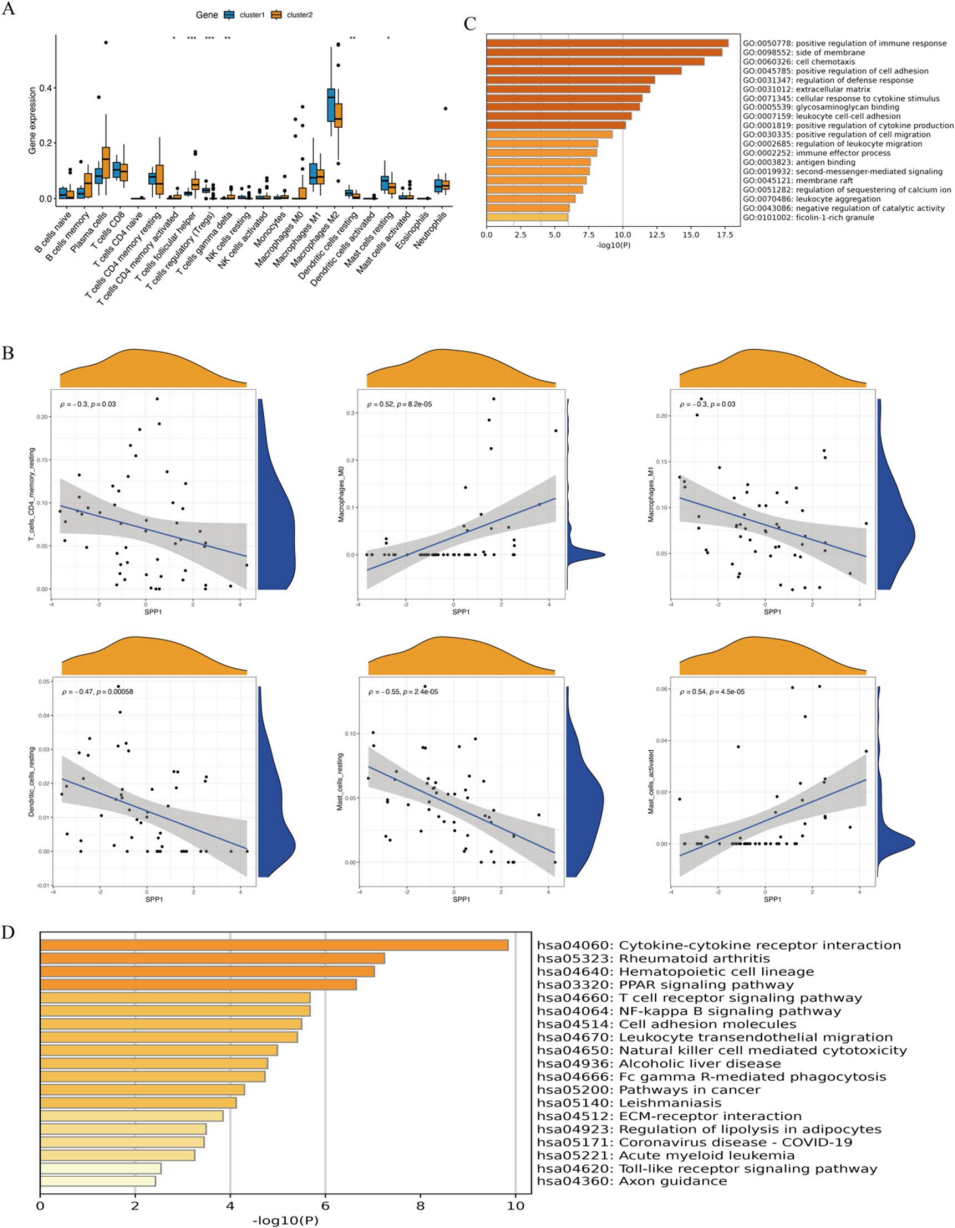
Immune characteristics of the two PANoptosis subtypes in RA. **A** CIBERSORT analysis reveals the differences in immune characteristics between cluster A and cluster B subtypes. **B** Correlation analysis between the feature gene SPP1 and immune cells. **C** GO enrichment analysis of differentially expressed genes between the two subtypes. **D** KEGG enrichment analysis of differentially expressed genes between the two subtypes

Next, we analyzed the differences in immune characteristics between the two subtypes of RA based on the all gene expression profile. Immune cell infiltration analysis revealed that Cluster 1 exhibited high expression of Tregs, resting dendritic cells, and resting mast cells. Cluster 2, on the other hand, showed high expression of activated CD4 memory T cells, follicular helper T cells, and gamma delta T cells (Fig. 6A). We validated the correlation between the SPP1 gene and immune cells and found a positive correlation between SPP1 and Macrophages M0 (R = 0.52, *P* = 8.2e − 05) and Mast cells activated (R = 0.54, *P* = 4.5e − 05). SPP1 was negatively correlated with T cells CD4 memory resting (R =  − 0.3, *P* = 0.03), Macrophages M1 (R =  − 0.3, *P* = 0.03), resting dendritic cells (R =  − 0.47, *P* = 0.00058), and resting mast cells (R =  − 0.55, *P* = 2.4e − 05) (Fig. 6B). We analyzed the differentially expressed genes between the two subtypes, with the parameter set as logfc > 1 and adjusted *p*-value < 0.05. We identified a total of 170 differentially expressed genes. GO enrichment analysis revealed that these genes were mainly enriched in processes such as positive regulation of immune response, cell chemotaxis, and positive regulation of cell adhesion (Fig. 6C). KEGG enrichment analysis showed that these genes were mainly involved in pathways such as cytokine-cytokine receptor interaction, hematopoietic cell lineage, PPAR signaling pathway, T cell receptor signaling pathway, and NF-kappa B signaling pathway (Fig. 6D).

**Fig. 7:**
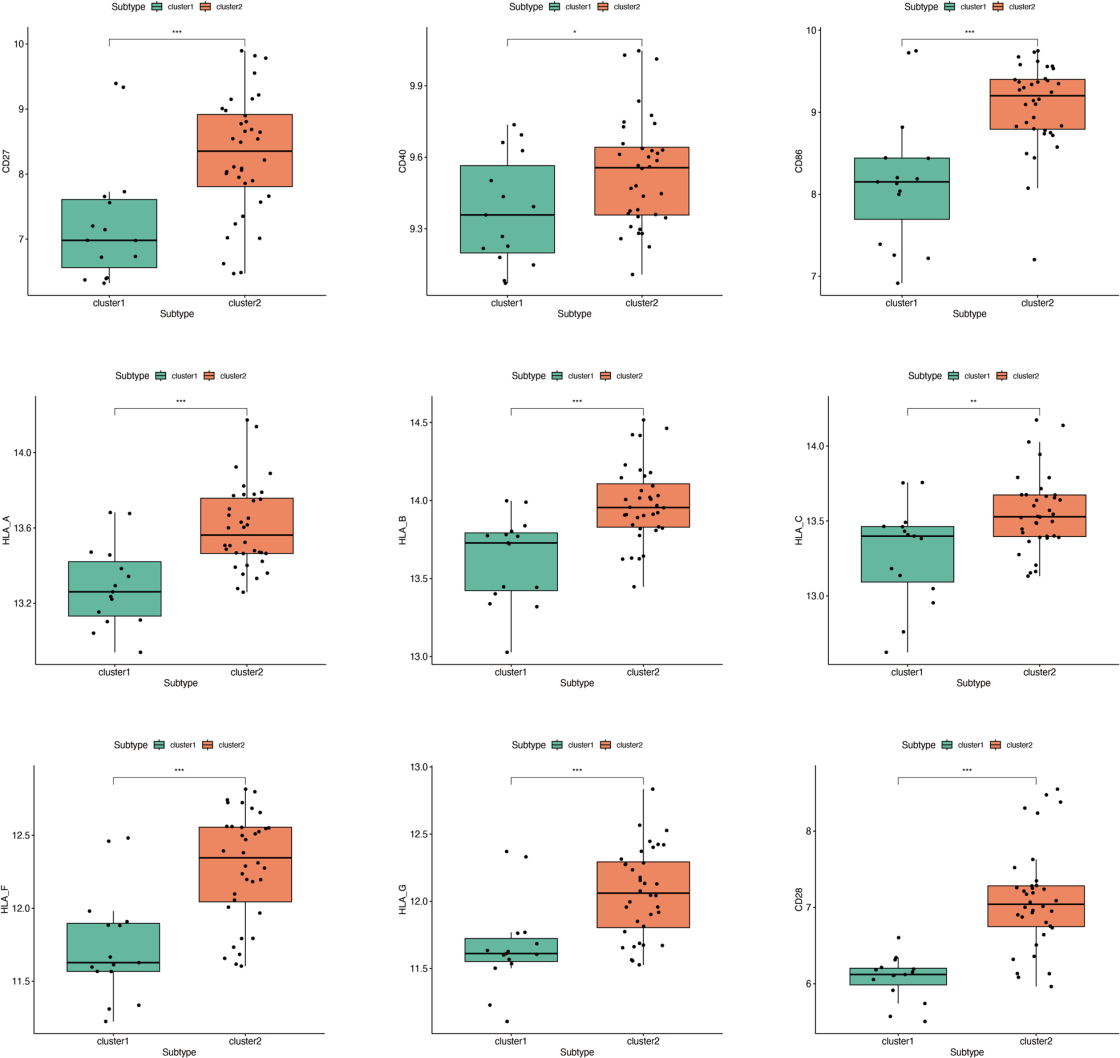
Immune checkpoint analysis of two PANoptosis subtypes in RA

We further analyzed the differences in common immune checkpoint expression between the two clusters and found that Cluster 2 exhibited better sensitivity to immune checkpoint therapy. Specifically, it was characterized by higher expression of CD28 (encoding T-cell-specific surface glycoprotein), CD40 (TNF Receptor Superfamily Member), CD27 (TNF receptor superfamily), CD86 (T-Lymphocyte Activation Antigen), HLA-A, HLA-B, HLA-C, HLA-G, and HLA-F (HLA Class I Histocompatibility Antigen) (Fig. 7).

### Construction and predictive value of PANoptosis score in patients with RA

**Fig. 8:**
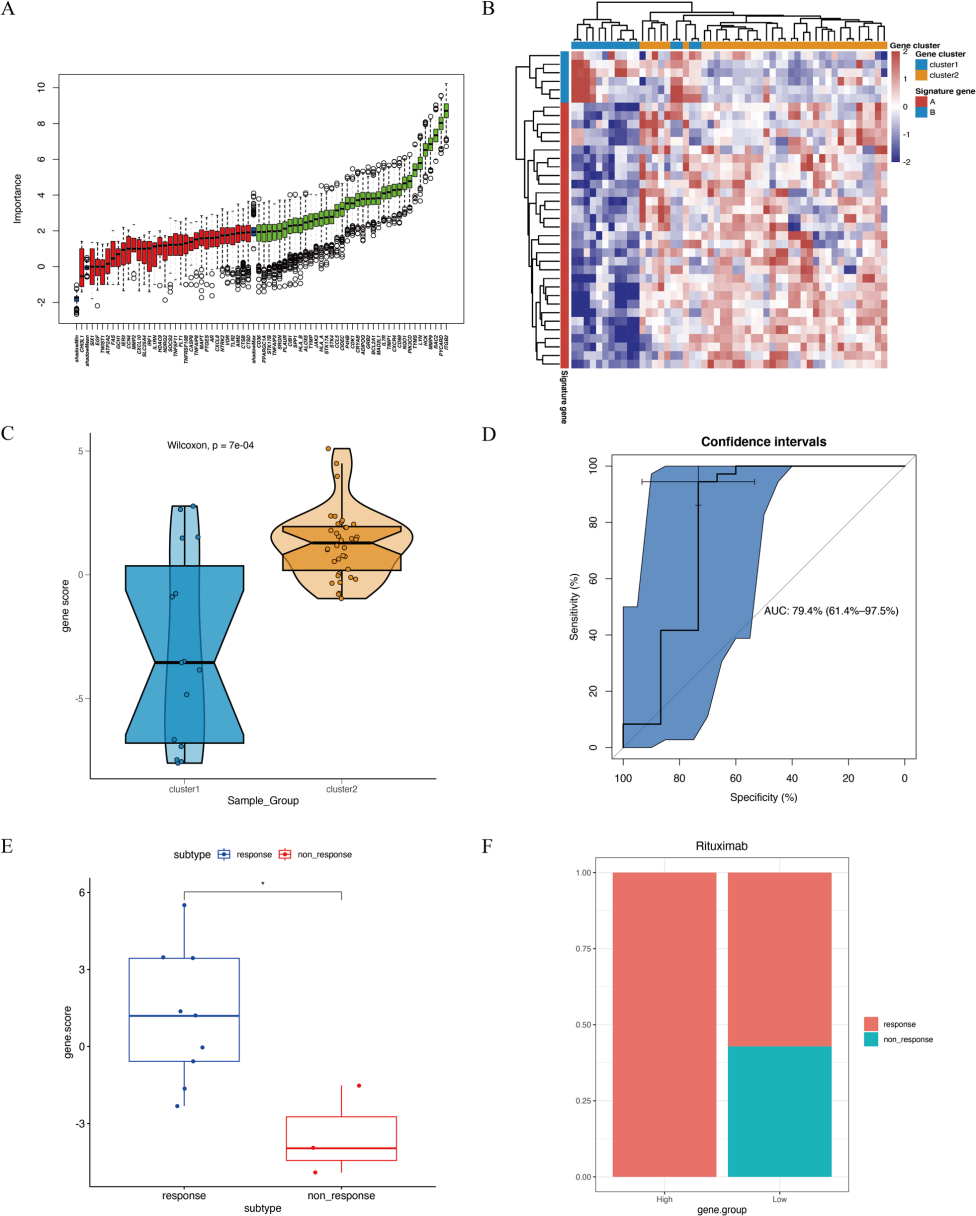
Illustrates the construction of the PANoptosis score model for RA patients. **A** Boruta algorithm used for gene dimensionality reduction. **B** Heatmap showing the expression of PANoptosis score genes between the two RA subtypes. **C** Boxplot showing the difference in PANoptosis scores between the two subtypes. **D** ROC curve for predicting subtypes using the PANoptosis score. **E** Boxplot showing the difference in PANoptosis scores between responders and non-responders to Rituximab treatment. **F** Rituximab treatments respond to the RA subtypes

To evaluate the pan-apoptotic modification pattern in RA patients, we utilized the Boruta algorithm to reduce dimensionality based on 68 differentially expressed PANoptosis genes. Using the PCA algorithm, we defined two scores: Score A, consisting of 31 genes specifically associated with disease, and Score B, consisting of 6 genes specifically associated with a negative correlation to disease (Fig. 8A,B). We calculated the PANoptosis score for each RA patient. Rank sum test analysis revealed that cluster 2 had a higher PANoptosis score compared to cluster 1 (*p* = 7e − 04) (Fig. 8C). We used the PANoptosis score to predict different subtypes, and the area under the ROC curve indicated its high predictive value (AUC = 0.794) (Fig. 8D). The sensitivity and specificity of this score model were 0.73 and 0.94, respectively. We applied the PANoptosis score model to accurately assess the response to drugs in different subtypes. Using the median of the PANoptosis score as a threshold, patients were categorized into high or low PANoptosis score groups. To further understand the impact of the PANoptosis score on predicting drug response, we selected four independent synovial tissue sequencing samples from RA patients receiving drug treatments. In the GSE24742 dataset, 12 patients were treated with Rituximab. We tested the difference in PANoptosis between responders and non-responders to Rituximab and found that responders had higher PANoptosis scores (Fig. 8E, F). In GSE172188, 10 RA patients received Abatacept treatment. In GSE15602, 11 RA patients received Adalimumab treatment. In GSE45867, 12 RA patients received Tocilizumab treatment, while 8 patients received Methotrexate treatment. However, these datasets did not reveal any differences in PANoptosis scores between responders and non-responders (Supplementary Figure S2). In summary, we found that RA patients with high PANoptosis scores showed a better response to Rituximab drug treatment.

## Discussion

The etiology of RA is multifaceted, encompassing genetic, environmental, and immune components [19]. Emerging evidence indicates the significance of PANoptosis-related genes in the pathogenesis of RA. Nevertheless, limited research has investigated the association between RA and PANoptosis through transcriptomics integration. This study primarily examined the expression patterns of PANoptosis genes in RA, employing differential expression analysis, WGCNA, and diverse machine learning algorithms as fundamental screening methodologies to identify accurate and cost-effective diagnostic biomarkers for PANoptosis in RA patients. In this study, we conducted clustering analysis utilizing differentially expressed PANoptosis genes to identify two distinct PANoptosis subtypes in RA. Furthermore, we characterized the differences in the immune microenvironment between these subtypes and developed a PANoptosis subtype score. Additionally, we assessed the sensitivity of the different subtypes to various drug treatments. Our investigation also highlighted SPP1 as a potential significant biomarker in RA.

We performed enrichment analysis of GO and KEGG on 30 overlapping genes, revealing their involvement in NF-kappaB transcription and regulation of cysteine-type endopeptidase activity, NF-kappaB transcription, regulation of cysteine-type endopeptidase activity involved in the apoptotic signaling pathway, and Toll-like receptor binding. NF-κB, a nuclear transcription factor, plays a crucial role in cellular processes including cell proliferation, apoptosis, and differentiation [20]. Research indicates that NF-κB downregulates miR-1276 expression by binding to its promoter, consequently enhancing the expression of microphthalmia-associated transcription factor and facilitating osteoclast differentiation [21]. Furthermore, FOXC1-mediated TRIM22 governs the excessive proliferation and inflammation of fibroblast-like synoviocytes implicated in rheumatoid arthritis via the NF-κB signaling pathway [22]. The inhibition of cell proliferation and inflammation in RA synovial fibroblasts through the overexpression of miR-27a-3p, which targets Toll-like receptor 524, presents promising implications for the treatment of RA [23]. These pieces of evidence suggest a potential value of targeting the pan-apoptotic pathway for treating RA patients.

Previous studies have demonstrated the efficacy of machine learning algorithms in identifying sensitive diagnostic biomarkers for different diseases [24, 25]. In this particular study, we employed machine learning techniques to identify diagnostic biomarkers associated with the pan-apoptotic pathway in RA. Four machine learning algorithms, Boruta, LASSO, SVM-REF, and RF, were employed to select key genes, and SPP1 was confirmed to be an effective biomarker. SPP1, also referred to as osteopontin, is a prevalent extracellular matrix protein and pro-inflammatory cytokine that engages with integrin receptors on the cell surface, thereby promoting cell adhesion and communication [26]. The secretion of SPP1 by fibroblast-like synoviocytes promotes osteoclast formation through the PI3K/AKT signaling pathway in collagen-induced arthritis [27]. In a recent study, Alivernini et al. employed scRNA-seq technology to characterize macrophages in the synovial tissue of patients with RA. The researchers discovered that synovial macrophages expressing SPP1/osteocalcin were more abundant in active RA cases and exhibited a positive correlation with disease activity. These macrophages displayed elevated levels of cytoskeletal proteins and integrins, indicating a migratory phenotype [28]. Steven et al. conducted an evaluation on the impact of SPP1 on the advancement of RA and discovered a significant correlation between SPP1 rs11439060 and rs9138 variants and decreased serum OPN expression, suggesting their association with disease progression [29]. Additionally, previous literature has demonstrated that SPP1 secreted by RA synovial fibroblasts stimulates osteoclastogenesis through the PI3K/AKT signaling pathway [27]. These investigations emphasize the crucial role of the SPP1 gene in RA. Using differentially expressed pan-apoptotic genes as a basis, we have successfully identified two distinct subtypes of RA.

We have developed a PANoptosis score to assess the responsiveness of these subtypes to drug treatments by leveraging existing datasets. Our findings indicate that patients with a high PANoptosis score demonstrate heightened sensitivity to immune checkpoint therapy and exhibit favorable responses to Rituximab treatment. CD28 and CD226 have been identified as potential risk factors for inflammatory arthritis due to their involvement in T cell co-stimulation [30]. The simultaneous inhibition of ICOS and CD28 signaling, achieved through the use of inhibitors like Acazicolcept, has proven effective in reducing inflammation and slowing the progression of RA and psoriatic arthritis (PsA) [31]. Variants in the CD40 locus have also been discovered to impact the development of inflammatory diseases, including RA [32, 33]. Abatacept, on the other hand, directly targets B cells by decreasing the expression of CD80/CD86, thereby offering a potential therapeutic approach for treating B cell-mediated autoimmunity [34]. Furthermore, our study indicates that the presence of HLA-related immune checkpoints, characterized by elevated expression of HLA alleles in the high PANoptosis score group, may play a role in the development of inflammatory arthritis, including RA, spondyloarthritis, and systemic juvenile idiopathic arthritis [35]. Additionally, Rituximab, by specifically targeting the CD20 antigen and inducing apoptosis in B lymphocytes, has demonstrated promising effectiveness in treating antibody-mediated rheumatoid arthritis [36, 37]. Consequently, the pan-apoptotic gene-based score holds the potential to identify distinct subtypes and offer valuable insights for the management of RA.

## Conclusion

In summary, our study employed a range of machine learning algorithms to identify the PANoptosis biomarker SPP1 in RA patients, which was subsequently validated in an independent external dataset, demonstrating its elevated expression. Additionally, utilizing pan-apoptotic gene expression profiles, we have discerned two distinct subtypes of RA and have elucidated the variances in immune cells, immune checkpoints, and immune pathways between these subtypes. Furthermore, we have developed a PANoptosis score model that demonstrates promising predictive capabilities in distinguishing between these subtypes and may hold clinical significance in guiding medication decisions. These findings suggest that the identified genes may possess a pivotal role in the pan-apoptotic pathway in RA. Our study contributes to a more comprehensive comprehension of PANoptosis in RA, although further evidence is necessary to validate our findings.

### Supplementary Information

**Additional file 1: Figure S1.** Validation of SPP1 differentially expressed genes in the independent datasets; (A) GSE55235. (B) GSE12021. (C) GSE55457. **Figure S2.** Drug treatments respond to the RA subtypes. (A) GSE172188. (B) GSE15602. (C) GSE45967.

## Data Availability

The datasets supporting the conclusions of this article are available in the GEO repository (https://www.ncbi.nlm.nih.gov/geo/).
