# Supplementary material for: Machine learning and molecular subtype analyses provide insights into PANoptosis-associated genes in rheumatoid arthritis
Source: Arthritis Res Ther. 2023 Dec 1;25:233. doi: 10.1186/s13075-023-03222-4 (PMC10691119; doi:10.1186/s13075-023-03222-4)
Supplement: Supplementary file 1 — Additional file 1: Figure S1. Validation of SPP1 differentially expressed genes in the independent datasets; (A) GSE55235. (B) GSE12021. (C) GSE55457. Figure S2. Drug treatments respond to the RA subtypes. (A) GSE172188. (B) GSE15602. (C) GSE45967. [file 13075_2023_3222_MOESM1_ESM.docx]

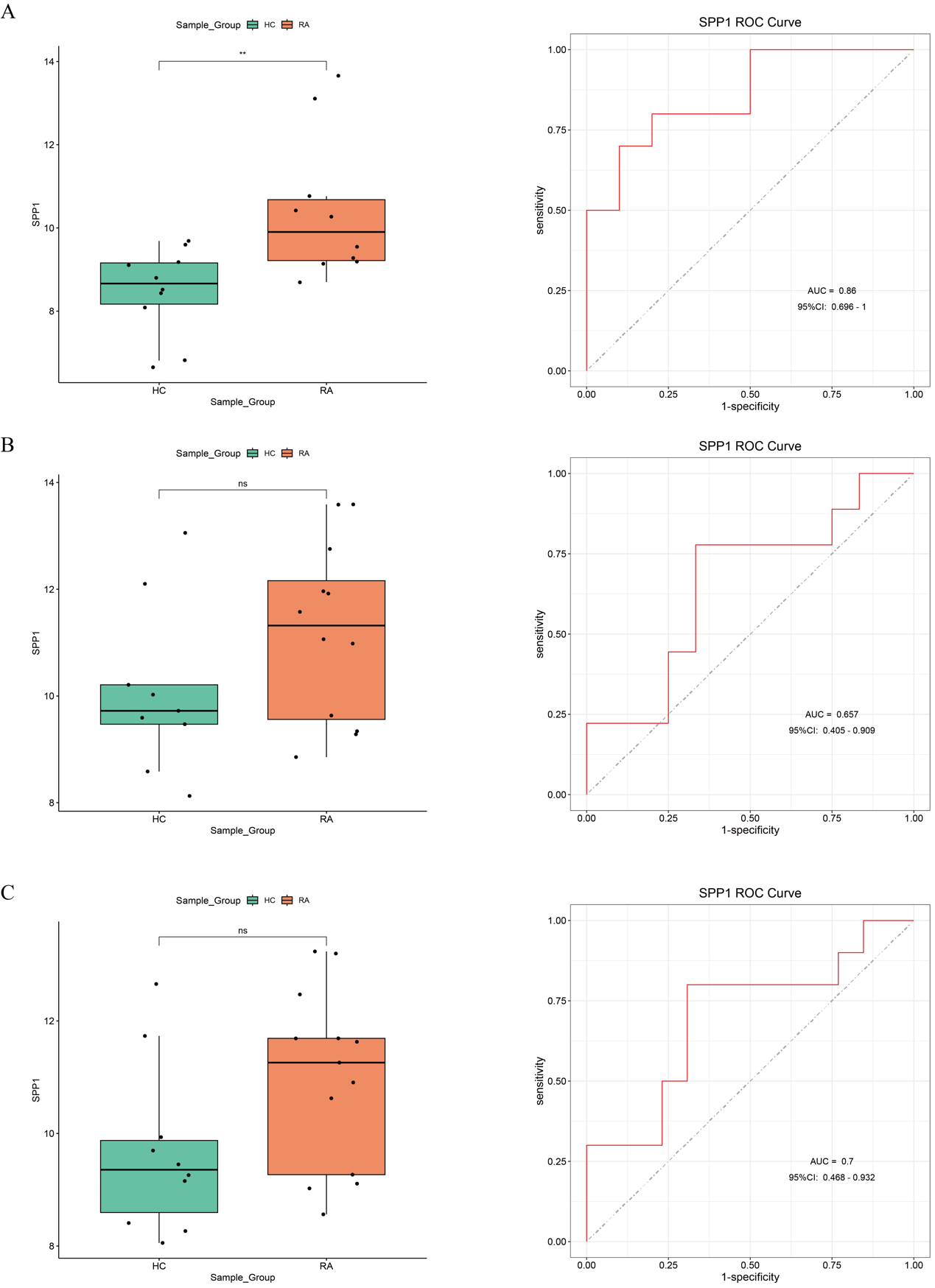


Figure S1 Validation of SPP1 differentially expressed genes in the independent datasets; (A) GSE55235. (B) GSE12021. (C) GSE55457.


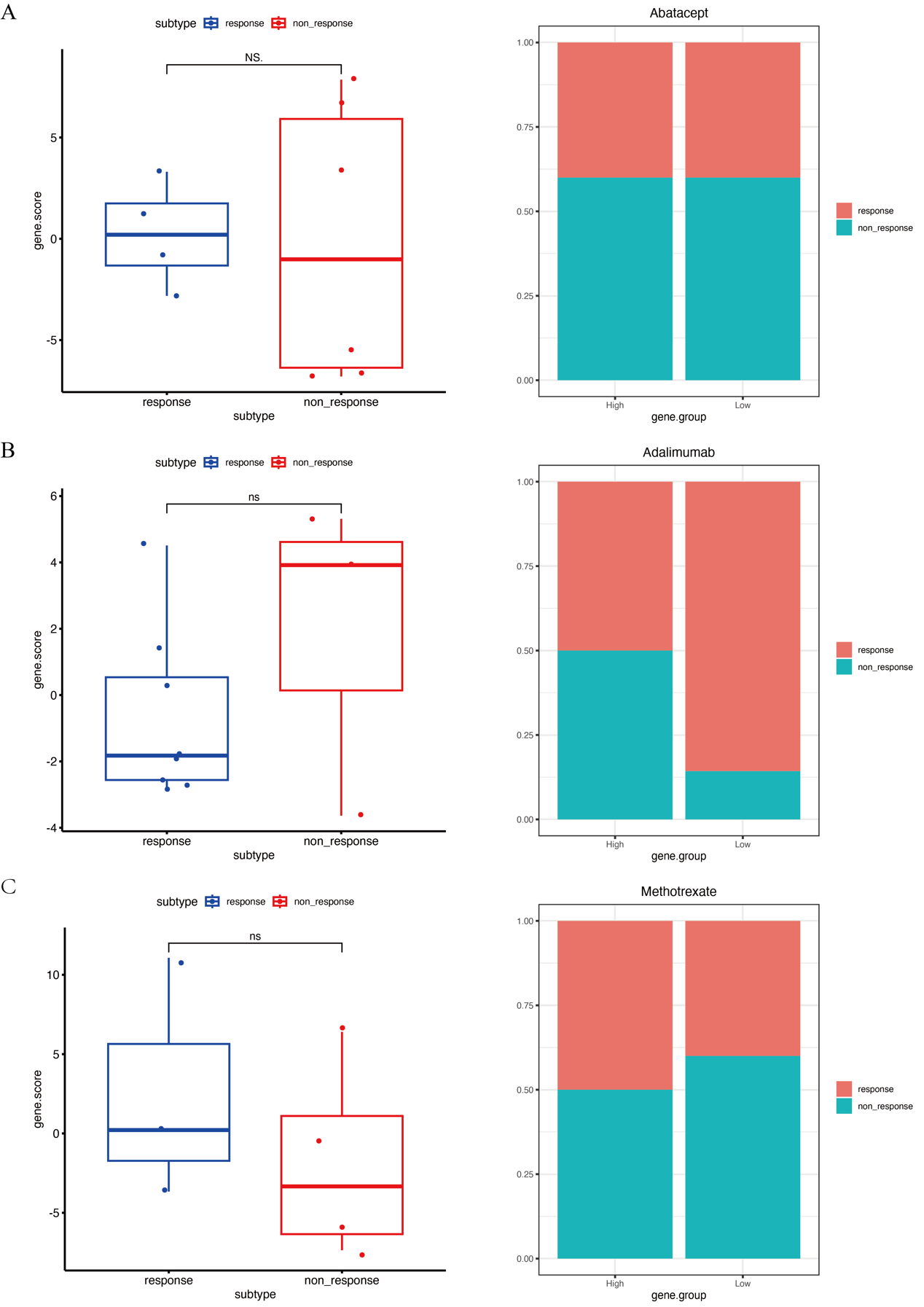


Figure S2 Drug treatments respond to the RA subtypes. (A) GSE172188. (B) GSE15602. (C) GSE45967.
